# Activity of Bacteriophages in Removing Biofilms of *Pseudomonas aeruginosa* Isolates from Chronic Rhinosinusitis Patients

**DOI:** 10.3389/fcimb.2017.00418

**Published:** 2017-09-22

**Authors:** Stephanie A. Fong, Amanda Drilling, Sandra Morales, Marjolein E. Cornet, Bradford A. Woodworth, Wytske J. Fokkens, Alkis J. Psaltis, Sarah Vreugde, Peter-John Wormald

**Affiliations:** ^1^Department of Surgery—Otolaryngology, Head and Neck Surgery, The Queen Elizabeth Hospital Woodville South, SA, Australia; ^2^AmpliPhi Australia Brookvale, NSW, Australia; ^3^Department of Otorhinolaryngology, Academic Medical Center, University of Amsterdam Amsterdam, Netherlands; ^4^Department of Otolaryngology-Head and Neck Surgery, University of Alabama at Birmingham Birmingham, AL, United States

**Keywords:** bacteriophage, *Pseudomonas aeruginosa*, chronic rhinosinusitis, cystic fibrosis, biofilm, multidrug resistant

## Abstract

**Introduction:** *Pseudomonas aeruginosa* infections are prevalent amongst chronic rhinosinusitis (CRS) sufferers. Many *P. aeruginosa* strains form biofilms, leading to treatment failure. Lytic bacteriophages (phages) are viruses that infect, replicate within, and lyse bacteria, causing bacterial death.

**Aim:** To assess the activity of a phage cocktail in eradicating biofilms of *ex vivo P*.aeruginosa isolates from CRS patients.

**Methods**: *P*. *aeruginosa* isolates from CRS patients with and without cystic fibrosis (CF) across three continents were multi-locus sequence typed and tested for antibiotic resistance. Biofilms grown *in vitro* were treated with a cocktail of four phages (CT-PA). Biofilm biomass was measured after 24 and 48 h, using a crystal violet assay. Phage titrations were performed to confirm replication of the phages. A linear mixed effects model was applied to assess the effects of treatment, time, CF status, and multidrug resistance on the biomass of the biofilm.

**Results:** The isolates included 44 strain types. CT-PA treatment significantly reduced biofilm biomass at both 24 and 48 h post-treatment (*p* < 0.0001), regardless of CF status or antibiotic resistance. Biomass was decreased by a median of 76% at 48 h. Decrease in biofilm was accompanied by a rise in phage titres for all except one strain.

**Conclusion:** A single dose of phages is able to significantly reduce biofilms formed *in vitro* by a range of *P*.aeruginosa isolates from CRS patients. This represents an exciting potential and novel targeted treatment for *P. aeruginosa* biofilm infections and multidrug resistant bacteria.

## Introduction

Chronic rhinosinusitis (CRS) is an inflammatory condition of the nose and paranasal sinuses, persisting for 12 weeks or longer. Bacterial biofilms have been implicated in recalcitrant CRS and increase the tolerance of bacteria to antibiotics through numerous mechanisms. These include metabolic heterogeneity of the bacteria within the biofilm, enzymatic deactivation, anionic charges due to extracellular DNA within the biofilm matrix, and changes in gene expression (de la Fuente-Nú-ez et al., 2013). Biofilms have been found on the sinonasal mucosa of up to 54% of CRS sufferers, compared to 8% of control patients (Chen et al., 2012). Furthermore, multiple studies have noted a higher prevalence of biofilms in patients who are undergoing revision surgery (Psaltis et al., 2007; Chen et al., 2012). In particular, the presence of biofilm-forming *Pseudomonas aeruginosa* strains has been associated with poor resolution of symptoms and signs of CRS following endoscopic sinus surgery (Bendouah et al., 2006).

*P. aeruginosa* has been identified in the sinuses of 9% of CRS patients, and is associated with poorer quality of life measured by disease severity scores, such as the Visual Analogue Scale (VAS) and Sinonasal Outcome Test-22 (SNOT-22) (Cleland et al., 2013, 2016). *P. aeruginosa* sinus infections also commonly afflict patients with cystic fibrosis (CF), with the species being identified in sinus cultures of up to 49% of CF patients with CRS (Rasmussen et al., 2012). Moreover, *P. aeruginosa* is intrinsically resistant to many classes of antibiotics, and acquired antibiotic resistance is increasing worldwide. New therapeutic strategies are therefore required to combat these difficult to treat bacterial infections in the context of CRS.

Bacteriophages are viruses that infect bacteria. Lytic bacteriophages are able to lyse their host bacterium, after replicating themselves within the host bacterium. Lysis of the host not only kills the bacterium, but also releases the progeny copies of the phage for re-infection of other bacteria (Guttman et al., 2004). Bacteriophages are species specific, and so can be used to target pathogenic bacteria, without disturbing non-harmful commensal bacteria (Hanlon, 2007). Bacteriophages are also able to penetrate bacterial biofilms (Vilas Boas et al., 2016).

These characteristics make bacteriophages an attractive non-antibiotic therapy for treating bacterial biofilms in CRS. This study aims to assess the activity of a cocktail (mixture) of four *P. aeruginosa* bacteriophages (CT-PA) in removing *ex vivo* biofilms formed by PA isolates from CRS patients, both with and without CF. The prevalence of antibiotic resistance in these clinical isolates was also assessed.

## Methods

### Bacterial strains and growth conditions

This study was approved by the Human Research Ethics Committee of The Queen Elizabeth Hospital, Adelaide, South Australia.

*P. aeruginosa* strains were isolated by an independent pathology laboratory (Adelaide Pathology Partners, Adelaide, South Australia) from endoscopically-guided sinus swabs, from patients who met the European Position Paper on Rhinosinusitis and Nasal Polyps (EPOS) 2012 criteria for chronic rhinosinusitis (Fokkens et al., 2012). Clinical *P. aeruginosa* isolates of patients with CF were kindly donated by the Department of Otorhinolaryngology, Academic Medical Centre (Amsterdam, Netherlands) and *P. aeruginosa* sinus isolates from CRS patients with and without CF were kindly donated by the Department of Otolaryngology-Head and Neck Surgery, University of Alabama at Birmingham (Birmingham, AL). *P. aeruginosa* isolates were stored in 25% glycerol in nutrient broth at −80°C. *P. aeruginosa* laboratory reference strain ATCC 15692 (PAO1) was obtained from American Type Culture Collection (Manassas, VA, USA) as a control for phage sensitivity and biofilm assays. Isolates were plated from frozen glycerol stocks onto 1.5% nutrient agar, and broth cultures were grown in nutrient broth. Agar plates and broth cultures were incubated at 37°C.

### Bacteriophage cocktail

Stocks of 4 anti-*P. aeruginosa* bacteriophages (Pa 193, Pa 204, Pa 222, Pa 223), as well as heat-inactivated stocks, were supplied by AmpliPhi Biosciences (Brookvale, New South Wales, Australia). Pa 193 and Pa 204 are of the Myoviridae family, and Pa 222 and Pa 223 are of the Podoviridae family. All 4 phages have been characterized as strictly lytic by genome sequencing (unpublished data). Prior to each assay, the stock suspension of each bacteriophage was titrated against a selected *P. aeruginosa* bacterial strain using the soft agar overlay small drop assay, as described below. Equal concentrations of each bacteriophage were combined to form the bacteriophage cocktail (CT-PA).

### Multi-locus sequence typing

The protocol for multi-locus sequence typing (MLST) of *P. aeruginosa* isolates has been described by Curran et al (Curran et al., 2004). For each strain, a single colony was used to inoculate 5 mL of nutrient broth, and the culture was grown overnight on a shaker at 37°C. DNA extraction from a 1 mL aliquot of the overnight culture was performed using the DNeasy Blood and Tissue DNA extraction kit (Qiagen, Hilden, Germany), following the protocol for Gram negative bacteria recommended by the manufacturer. PCR of the 7 MLST loci was performed using the Taq PCR kit (New England Biolabs, Ipswich MA, USA) using the protocol and primers described by Curran et al (GeneWorks, Adelaide, South Australia). PCR products were purified using the QIAquick PCR Purification kit (Qiagen, Hilden, Germany), following the manufacturer's protocol. Sanger sequencing was performed on the purified PCR products by two external laboratories (SA Pathology and Australian Genome Research Facility, Adelaide, South Australia). Sequences were checked for base miscalls, and contigs were assembled using GeneStudio Professional, version 2.2.0.0 (GeneStudio Inc.). The sequence of each locus was matched to the allele sequences in the *P. aeruginosa* MLST database (pubmlst.org/paeruginosa/) and assigned the corresponding allele number. The profile of alleles for each isolate was then matched to the sequence type profiles in the MLST database.

### Phylogenetic and burst analysis

Phylogenetic analysis of the clinical isolates and laboratory reference strain PAO1 were conducted using MEGA version 6.06 (Tamura et al., 2013). MLST sequence alleles for each of the 7 loci were aligned using the MUSCLE programme in MEGA (Edgar, 2004). The concatenated alignments were used to generate dendrograms using the Neighbor-Joining method with bootstrapping analysis (2,000 replicates) (Felsenstein, 1985). Evolutionary distances were computed using the Maximum Composite Likelihood method, with ambiguous positions removed for each sequence pair (Tamura et al., 2004). Outgroup sequences were identified through BLAST searching for sequences of sufficient homology from other *Pseudomonas* species. eBURST version 3 (eburst.mlst.net) was used to identify clonal complexes and BURST groups (Feil et al., 2004). Bootstrapping analysis with 2000 replicates was used for all BURST analyses.

### Minimum inhibitory concentration (MIC) assays

Resistance to commonly used antibiotics was determined using broth microdilution minimum inhibitory concentration (MIC) assays, as described by Wiegand et al. (2008). Antibiotics tested were: gentamicin, ciprofloxacin, ceftazidime, piperacillin, and amikacin, obtained from Sigma-Aldrich (Castle Hill, NSW, Australia). Isolates were designated as being sensitive, resistant, or having intermediate sensitivity to the antibiotics based on Clinical and Laboratory Standards Institute (CLSI) cut-offs.

### Sensitivity to CT-PA phages and enumeration of phage

The ability of the 4 phages to lyse each bacterial isolate was tested using the spot test described by Mazzocco et al, with a drop size of 5 μL (Mazzocco et al., 2009). All plaque assays were performed in duplicate. Enumeration of phage in stocks using the small drop plaque assay system was performed prior to each assay, with a concentration of 10^8^ PFU/mL used for isolate sensitivity assays. A selected reference bacterial strain was used for titration of each of the 4 different phage stocks. The small drop plaque assay was also used to assess the phage concentration in the liquid contents of the biofilm assay wells, at 48 h after treatments were applied.

### Biofilm assay

The microtitre dish biofilm formation assay as described by O'Toole was used to assess the ability of CT-PA to eradicate *P. aeruginosa* biofilms *in vitro* (O'Toole, 2011). A 1.0 McFarland unit suspension in 0.45% saline of the isolate was diluted into 10 times the volume of nutrient broth (Oxoid, Hants, UK), and gently mixed by inversion. 150 μL/well of the resulting suspension was plated into a clear polystyrene 96-well plate (Greiner Bio-One, Kremsmünster, Austria). Wells adjacent to the edge of the plate were filled with 180μL sterile PBS as a sterility control. The plate was then incubated for 48 h on a gyratory mixer at 37°C. After 48 h, the liquid contents of the bacterial wells were gently aspirated, followed by washing twice with sterile PBS to remove any remaining planktonic cells. Treatments included each of the 4 phages and the phage cocktail in nutrient broth at concentrations of 10^7^ and 10^8^ PFU/mL, as well as equivalent volumes of heat-inactivated stocks of the 4 phages, with PBS as negative control and 2.5% cetylpyridinium chloride (Sigma-Aldrich, St Louis, MO, USA) as a positive control. 180 μL of each treatment was plated in quadruplicate and biofilms were assessed at 24 and 48 h after treatment. Forty-eight hours treatment plates had 50 μL/well nutrient broth replenished at 24 h. At the designated time point (either 24 or 48 h after treatment), liquid contents of the wells were transferred into a fresh plate for post-treatment phage titration. The biofilm plates were gently washed twice with sterile PBS, and then stained with 190 μL/well 0.5% crystal violet (Sigma, St Louis, MO, USA) for 30 min. The stained plates were rinsed by two rounds of gentle immersion into distilled water, and left to dry overnight. The crystal violet stain was eluted by application of 200 μL/well 30% acetic acid (Chem-Supply, Adelaide, South Australia) and the plate was incubated at room temperature for 30 min. Absorbance at 595 nm was measured for each well using the Fluostar Optima microplate reader (BMG Labtech, Ortenberg, Germany), with 200 μL 30% acetic acid in unstained wells used as blanks.

### Statistics

Linear mixed effects models were applied to assess the effects of group, time point, CF status and multidrug resistance on absorbance (A595) data from the microtitre plate biofilm formation assay. A595 values were log transformed prior to analysis due to violations of the distributional assumptions of linear regression. Pairwise, *post-hoc* comparisons for group were assessed at *p* < 0.010 due to the large number of comparisons made. The data were analyzed using SAS v9.4 (SAS Institute Inc., Cary, NC, USA).

## Results

### Multiple MLST sequence types identified amongst CRS clinical isolates

In total, 47 *P. aeruginosa* isolates were collected from the upper and lower airways from 44 patients suffering from CRS and/or CF in 3 continents. These included 19 isolates from the upper and lower respiratory tracts of patients with CF (Amsterdam, Netherlands), 7 sinus isolates from CRS patients with and without CF (Birmingham, AL, USA), and 21 sinus isolates from CRS patients with and without CF (Adelaide, Australia). Isolate characteristics are displayed in Table 1. MLST revealed 44 distinct sequence types. Isolate MLST profiles and burst groups are detailed in Supplementary Table 1. One clinical isolate from a CF patient in the Netherlands was of the same sequence type as PA01. Two Australian non-CF isolates shared an identical sequence type (348), and another sequence type (274) was shared by one isolate from each of Australia, the United States, and the Netherlands.

**Table 1:** Characteristics of *P. aeruginosa* clinical isolates studied.

		**CF isolates**	**Non-CF isolates**	**Total**
Country of origin	Australia	2	19	21
	Netherlands	19	0	19
	USA	3	4	7
Total		24	23	47

**Figure 1:**
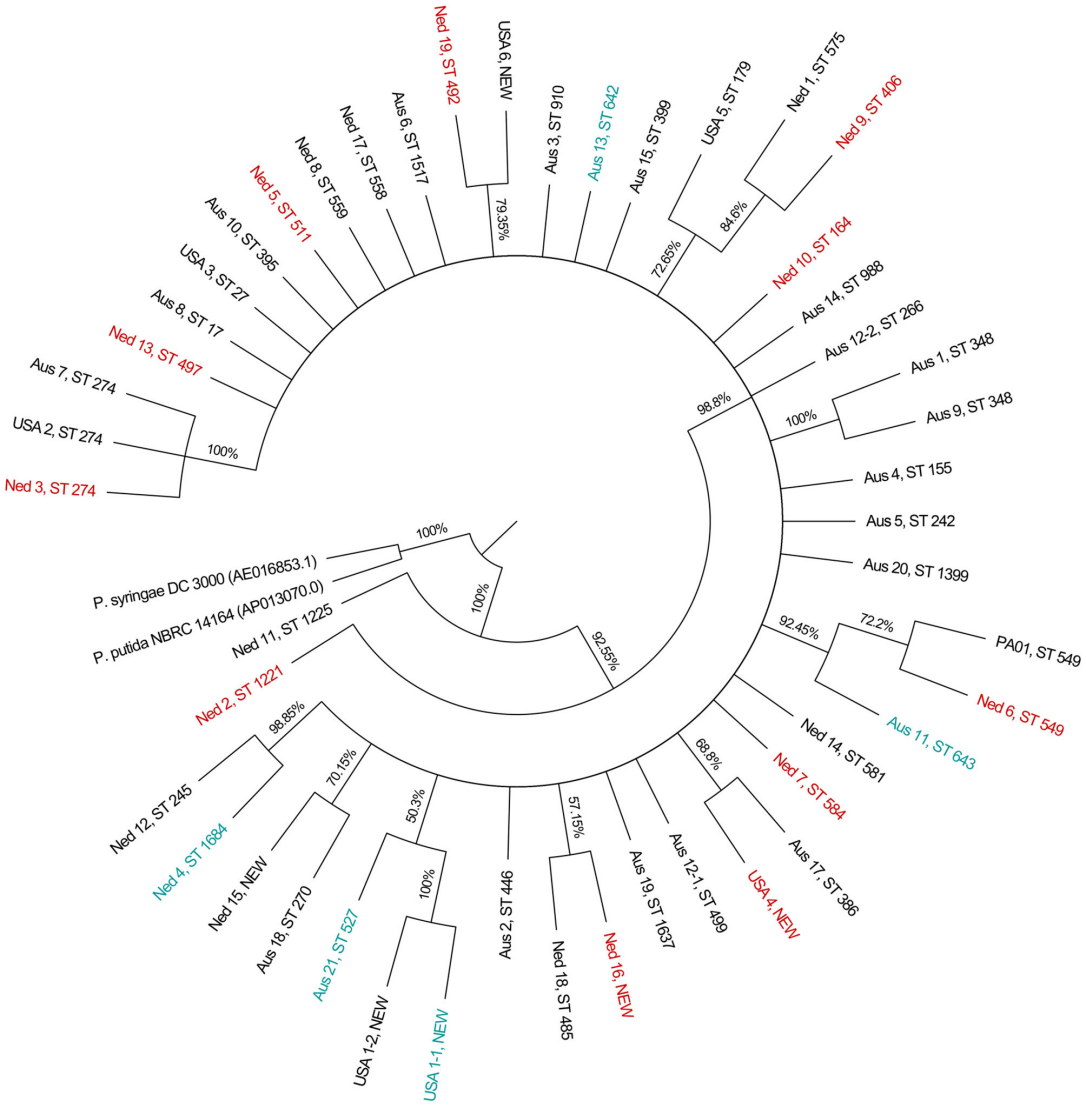
Condensed phylogenetic tree obtained using the Neighbor-Joining method. Phylogeny was inferred using the Neighbor-Joining method. The condensed topology after branches with bootstrap values of <50% have been collapsed into polytomies is shown. The percentages of replicate trees in which the associated taxa clustered together in the bootstrap test (2,000 replicates) are shown next to the branches. There were a total of 2,882 positions in the final dataset. Multidrug resistant isolates are labeled in red, and CT-PA phage cocktail resistant isolates are labeled in teal.

BURST analysis identified only one clonal complex, using the most stringent definition of sequence types sharing six or more alleles in common. The clonal complex consisted of two single locus variants isolated from the same CF patient. The phylogeny inferred by the Neighbor-Joining method is displayed in Figure 1.

### CT-PA host range in CRS clinical isolates

**Figure 2:**
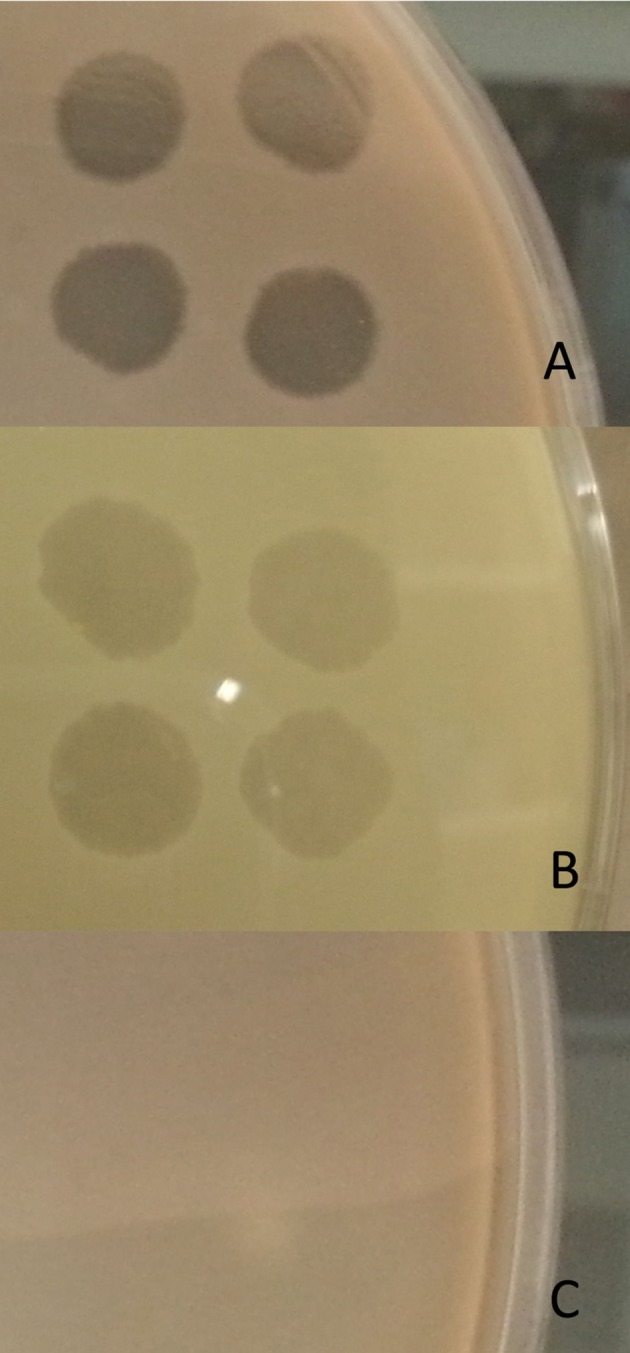
Spot test assay. Phage activity on the spot test assay was graded as: **(A)** Full activity—a completely clear (non-turbid) spot with no bacterial growth observed within the spot, **(B)** Partial activity—a turbid spot easily seen with the naked eye, or a clear spot containing isolated bacterial colonies, or **(C)** No activity—no easily discernible spot seen with the naked eye.

40/45 isolates (89%) were lysed by CT-PA, with partial or full activity spots seen on the small drop plaque assay. Examples of full and partial activity spots are shown in Figure 2. Two isolates could not be tested; one due to interference from plaques throughout the bacterial lawn including areas where the phage had not been applied (thought to be due to induction of a prophage), and the other due to poor growth in culture. When tested individually, 73, 53, 73, and 71% of isolates were sensitive to Pa 193, Pa 204, Pa 222, and Pa 223 respectively, as displayed in Table 2. 39/45 isolates (87%) were susceptible to 2 or more of the 4 phages.

**Table 2:** Host range of phages and phage cocktail in CRS clinical isolates.

***P. aeruginosa* isolate**	**Phage treatment**				
	**Pa 193**	**Pa 204**	**Pa 222**	**Pa 223**	**CT-PA**
PAO1 (reference strain)	++	++	++	0	++
Aus 1	0	0	++	++	++
Aus 2	Unable to test				
Aus 3	++	++	+	++	++
Aus 4	++	0	++	++	++
Aus 5	++	++	++	++	++
Aus 6	+	0	+	+	+
Aus 7	+	0	++	++	++
Aus 8	0	0	++	++	++
Aus 9	0	0	+	+	+
Aus 10	++	++	0	0	++
Aus 11	0	0	0	0	0
Aus 12	++	++	0	0	++
Aus 13	0	0	0	0	0
Aus 14	++	++	+	0	++
Aus 15	++	++	+	+	++
Aus 16	++	++	++	++	++
Aus 17	++	++	0	0	++
Aus 18	+	+	+	+	++
Aus 19	++	++	+	+	++
Aus 20	++	++	++	++	++
Aus 21	0	0	0	0	0
Ned 1	+	0	0	++	++
Ned 2	++	++	++	++	++
Ned 3	0	0	++	++	++
Ned 4	0	0	0	0	0
Ned 5	++	++	++	++	++
Ned 6	+	++	+	+	++
Ned 7	+	+	++	++	++
Ned 8	++	0	0	++	++
Ned 9	++	++	0	0	++
Ned 10	++	++	+	++	++
Ned 11	++	++	++	++	++
Ned 12	+	0	+	0	+
Ned 13	++	0	+	+	++
Ned 14	0	0	+	+	+
Ned 15	++	++	++	+	++
Ned 16	+	0	+	0	+
Ned 17	Unable to test				
Ned 18	0	0	++	+	++
Ned 19	+	0	0	0	+
USA 1-1	0	0	0	0	0
USA 1-2	0	0	+	++	++
USA 2	++	++	++	++	++
USA 3	++	++	++	++	++
USA 4-2	++	++	++	++	++
USA 5	++	++	+	+	++
USA 6	+	+	++	+	++
Total CI sensitive (%)	**33** (73)	**24** (53)	**33** (73)	**32** (71)	**40** (89)
Total CI resistant (%)	**12** (27)	**21** (47)	**12** (27)	**13** (29)	**5** (11)

*++, Full activity spots on small drop plaque assay*.

*+, Partial activity spots on small drop plaque assay*.

*0, No activity on small drop plaque assay*.

*CI, clinical isolates*.

*The bold values are the number (count) of isolates, as opposed to the percentage of isolates shown in parentheses*.

### Multidrug resistance in CF clinical isolates

Isolates that displayed sensitivity to CT-PA were tested for antibiotic resistance, using CLSI cut-offs for intermediate resistance and resistance. The number of isolates displaying resistance or intermediate resistance to the five antibiotics tested is shown in Table 3. Eleven isolates, all from CF patients, were multidrug resistant according to the definition proposed by Magiorakos et al. (2012).

**Table 3:** Antibiotic resistance of clinical isolates.

	**No. of resistant isolates** (%)		
	**CF isolates**	**Non-CF isolates**	**All isolates**
Gentamicin	**9** (43)	**1** (5)	**10** (25)
Amikacin	**10** (48)	**1** (5)	**11** (28)
Ciprofloxacin	**13** (62)	**2** (11)	**15** (38)
Ceftazidime	**12** (57)	**1** (5)	**13** (33)
Piperacillin	**3** (14)	**0** (0)	**3** (8)
Multidrug resistant	**11** (52)	**0** (0)	**11** (28)
Total no. of isolates tested	**21**	**19**	**40**

*The bold values are the number (count) of isolates, as opposed to the percentage of isolates shown in parentheses*.

### Biofilm reduction by CT-PA

**Figure 3:**
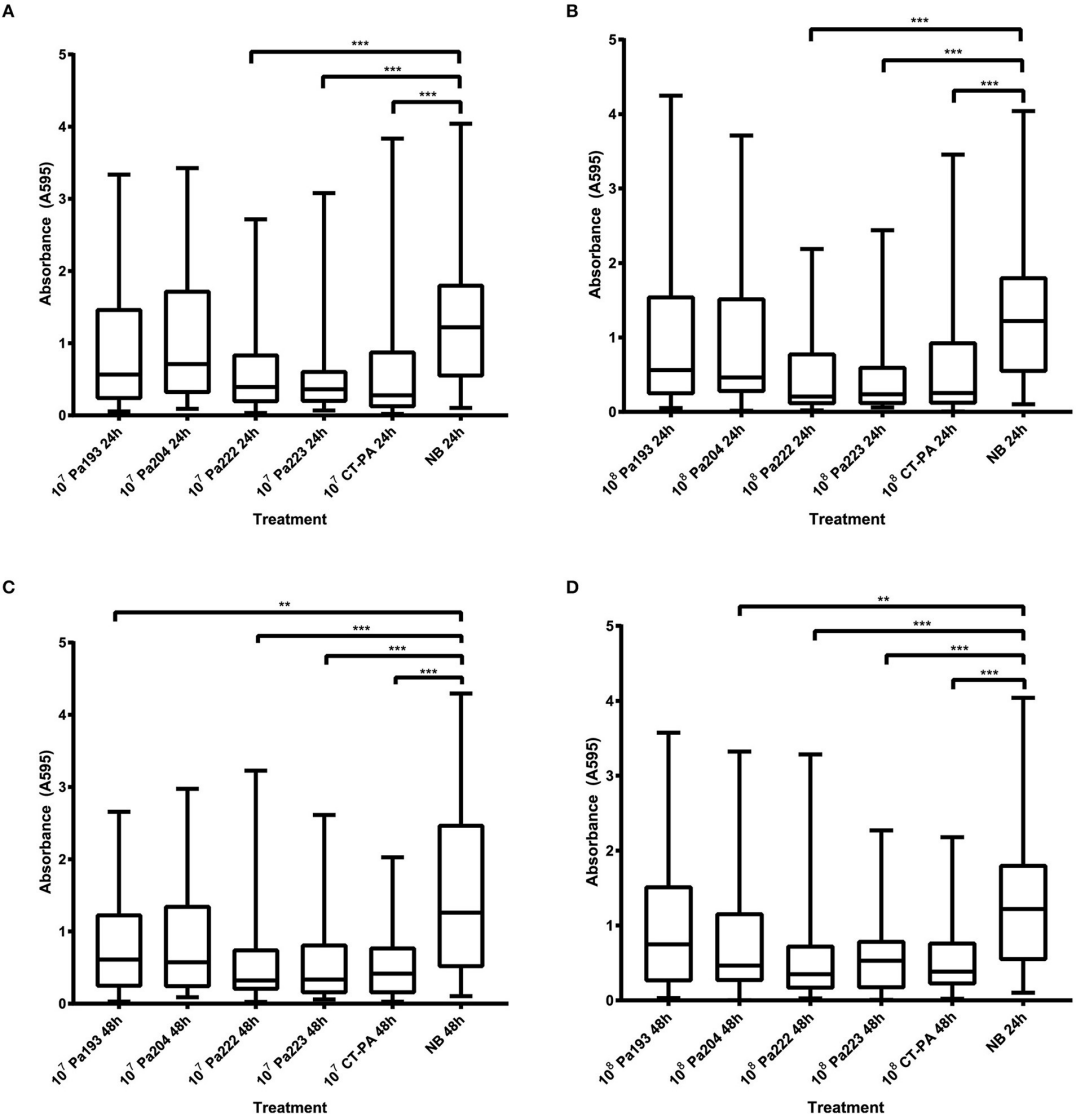
Absorbance at 595 nm of crystal violet-stained biofilms. Biomass of biofilms (of all 40 isolates and ATCC 15692) as measured by absorbance at 595 nm of crystal violet-stained biofilms after treatment with: **(A)** 10^7^ PFU/mL phage treatments or growth media control for 24 h, **(B)** 10^8^ PFU/mL phage treatments or growth media control for 24 h, **(C)** 10^7^ PFU/mL phage treatments or growth media control for 48 h, **(D)** 10^8^ PFU/mL phage treatments or growth media control for 48 h. Boxplot whiskers represent range (minimum to maximum values). (NB: nutrient broth; ^**^p < 0.01; ^***^p < 0.001).

All 40 isolates that displayed sensitivity to CT-PA on the spot test assay, as well as reference strain ATCC 15692, were tested using the microtitre dish biofilm assay. Absorbance readings from the crystal violet biofilm assays of all isolates at 24 and 48 h after treatment are displayed in Figure 3. Statistically significant reductions in biofilm biomass compared to the negative treatment control (nutrient broth) were seen with both 10^7^ and 10^8^ PFU/mL of CT-PA, Pa 222 and Pa 223 at both 24 and 48 h (*p* < 0.001). Significant reductions were also seen with 10^7^ PFU/mL Pa 193 and 10^8^ PFU/mL Pa 204 at 48 h (*p* < 0.01).

**Figure 4:**
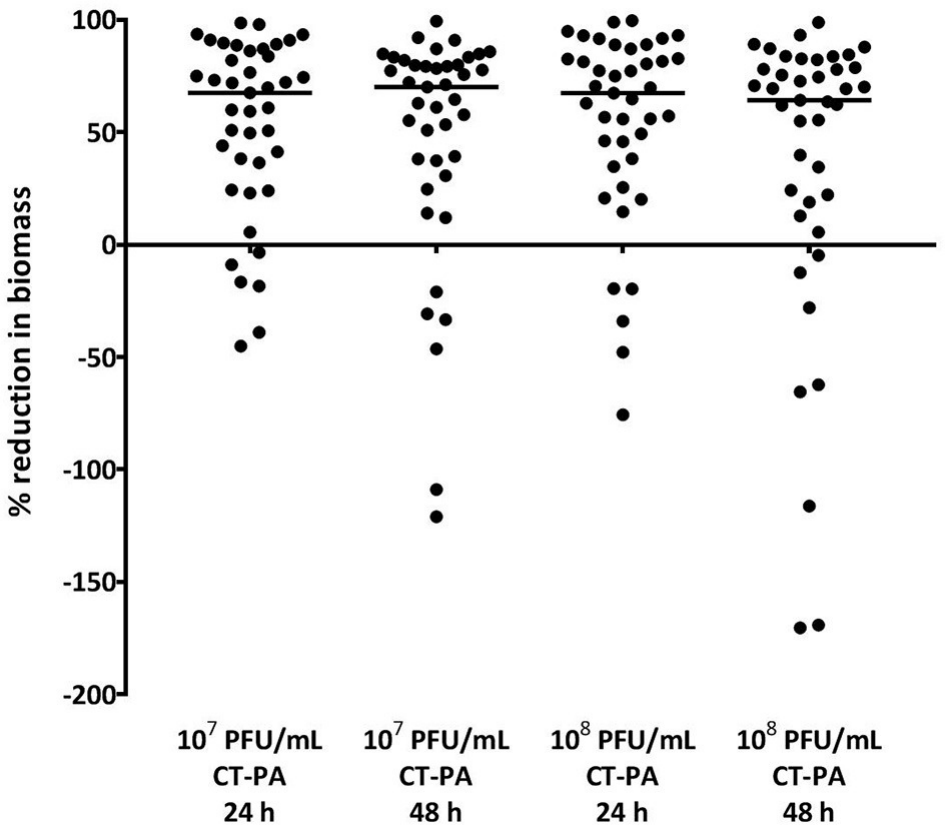
Percentage reduction in biofilm biomass with CT-PA treatment. Percentage reduction in biofilm biomass compared to growth media control after 24 and 48 h treatment with lower and higher concentrations of CT-PA. Dots represent the average of quadruplicate treatments, and horizontal lines indicate median values.

For all isolates tested, the median biofilm biomass reduction was 70% and 64% after 48 h treatment with the lower and higher concentrations of CT-PA, respectively. 34 (85%) and 32 (80%) of the clinical isolates, respectively showed a reduction in biomass compared to the negative treatment control at 48 h, as displayed in Figure 4. The median biomass reductions at the 24-h time point were similar for both concentrations of CT-PA at 67%. The maximum biomass reductions were 98.6 and 99.6% at 24 h, and 99.4 and 98.8% at 48 h, for the lower and higher concentrations, respectively.

Analysis of two-way interactions showed no statistically significant interactions between CF status or multidrug resistance and treatment effect.

### Post-treatment phage titres

For all except three of the 40 isolates tested, titres of two or more of the four phages had increased after 48 h treatment in the biofilm assay. This indicates successful phage infection and replication, and good complementation between the four phages comprising the CT-PA cocktail. Of the remaining three isolates, two showed an increase in titres of one phage, and one did not show an increase in any phage titres. Post-treatment phage titres for PAO1 and the 40 clinical isolates are displayed in Table 4.

**Table 4:** Post-treatment phage titres.

**Strain**	**Post-treatment phage titres (PFU/mL)**							
	**10^8^ PFU/mL treatment**				**10^7^ PFU/mL treatment**			
	**Pa 193**	**Pa 204**	**Pa 222**	**Pa 223**	**Pa 193**	**Pa 204**	**Pa 222**	**Pa 223**
PAO1	2.0E+10	3.0E+09	4.0E+10	1.0E+08	1.0E+10	2.0E+09	2.0E+10	1.0E+09
Aus 1	1.1E+08	5.7E+07	4.9E+07	1.0E+02	4.3E+06	4.0E+06	1.8E+08	2.0E+02
Aus 3	1.8E+10	9.2E+09	4.3E+09	3.9E+09	9.0E+09	2.9E+09	8.5E+08	6.5E+02
Aus 4	3.0E+08	9.0E+08	3.0E+08	1.0E+07	7.0E+08	4.0E+09	5.0E+06	3.0E+05
Aus 5	8.0E+10	2.4E+09	6.4E+08	2.0E+03	5.0E+09	1.3E+10	1.3E+09	1.2E+09
Aus 6	7.0E+07	2.0E+09	3.2E+09	2.1E+05	7.8E+06	7.6E+10	8.3E+09	2.1E+06
Aus 7	2.2E+09	1.0E+08	5.0E+08	4.0E+08	9.0E+09	1.0E+07	2.0E+08	1.0E+09
Aus 8	2.0E+03	8.0E+08	2.0E+03	1.0E+07	3.0E+09	2.0E+09	1.0E+08	5.0E+05
Aus 9	2.0E+07	4.8E+03	2.4E+09	1.3E+03	4.1E+04	6.0E+02	7.8E+08	2.1E+03
Aus 10	5.0E+07	1.0E+11	9.3E+08	1.2E+06	3.0E+06	7.0E+09	2.0E+09	8.0E+05
Aus 12	2.3E+09	2.6E+10	5.1E+09	3.8E+03	7.3E+08	8.2E+10	4.5E+09	2.7E+04
Aus 14	2.7E+08	5.3E+10	9.0E+07	4.8E+06	1.7E+08	2.4E+09	2.0E+06	3.9E+05
Aus 15	1.2E+11	3.0E+10	1.2E+10	5.1E+10	2.0E+11	3.0E+11	4.0E+10	2.0E+05
Aus 16	2.0E+10	1.0E+11	3.0E+09	2.0E+08	1.0E+10	1.0E+10	2.0E+07	2.0E+07
Aus 17	1.9E+10	5.2E+11	1.9E+10	9.5E+06	5.9E+10	1.5E+11	1.5E+10	5.2E+06
Aus 18	9.0E+08	1.0E+08	2.0E+10	2.5E+08	3.0E+07	2.0E+07	7.0E+10	1.0E+08
Aus 19	5.0E+08	2.0E+03	3.0E+10	2.0E+03	2.0E+03	2.0E+03	1.0E+09	2.0E+03
Aus 20	1.0E+10	4.0E+10	9.0E+07	1.0E+10	2.0E+10	2.0E+03	1.7E+07	5.0E+05
Ned 1	4.0E+07	5.3E+06	7.0E+05	5.9E+03	2.2E+06	7.1E+06	9.5E+03	6.4E+03
Ned 2	7.2E+09	1.1E+10	5.7E+07	1.5E+08	8.0E+09	2.0E+08	2.1E+07	8.0E+07
Ned 3	8.0E+07	1.9E+07	3.6E+08	1.5E+08	9.8E+05	2.0E+06	3.5E+07	5.7E+08
Ned 5	2.5E+10	2.1E+11	4.0E+07	7.7E+07	1.8E+10	1.1E+11	4.0E+07	9.0E+05
Ned 6	8.6E+09	2.4E+10	7.1E+08	1.8E+09	5.1E+09	1.7E+10	1.2E+08	5.2E+08
Ned 7	6.8E+10	1.5E+09	7.4E+07	8.4E+07	5.5E+10	1.6E+09	1.2E+07	2.6E+08
Ned 8	4.3E+10	2.2E+12	2.2E+09	2.1E+06	7.1E+10	2.4E+05	3.7E+13	1.5E+11
Ned 9	2.2E+10	2.3E+10	1.2E+07	5.5E+10	4.4E+10	1.0E+11	2.3E+06	1.6E+04
Ned 10	1.7E+08	1.1E+10	5.5E+06	7.0E+04	7.0E+07	1.7E+10	3.1E+05	2.0E+06
Ned 11	1.9E+09	3.9E+09	1.2E+09	8.5E+08	9.0E+09	6.9E+09	1.5E+09	2.4E+07
Ned 12	2.0E+08	1.0E+08	5.0E+09	1.7E+05	2.5E+06	1.0E+08	8.0E+09	4.0E+05
Ned 13	4.0E+09	3.2E+07	4.6E+08	1.0E+03	5.4E+09	4.6E+09	6.2E+08	0.0E+00
Ned 14	1.1E+09	5.3E+09	1.5E+08	1.8E+07	1.5E+09	4.5E+09	6.9E+07	4.3E+07
Ned 15	2.4E+11	1.9E+10	5.3E+09	4.9E+06	6.3E+10	1.5E+10	7.3E+09	5.6E+06
Ned 16	2.0E+11	3.4E+08	1.7E+08	5.1E+07	6.8E+10	3.0E+08	4.8E+08	8.0E+06
Ned 18	3.2E+07	1.4E+07	3.7E+09	1.8E+06	2.3E+06	1.4E+06	5.4E+09	2.7E+06
Ned 19	4.2E+08	1.2E+09	7.0E+07	2.2E+07	2.3E+06	6.2E+09	0.0E+00	6.8E+05
USA 1-2	3.0E+07	1.3E+07	3.0E+09	1.0E+06	6.0E+05	1.0E+07	3.0E+03	2.0E+06
USA 2	1.0E+09	2.0E+10	1.0E+09	1.0E+05	2.0E+10	3.0E+10	1.0E+11	6.0E+04
USA 3	1.0E+08	1.0E+12	1.0E+10	1.0E+01	1.0E+07	2.0E+06	8.0E+09	8.0E+03
USA 4-2	1.4E+09	9.0E+09	1.6E+09	1.0E+08	1.0E+10	2.0E+10	2.0E+09	1.0E+07
USA 5	1.0E+09	9.0E+09	2.0E+10	1.0E+05	1.0E+11	1.0E+09	5.0E+10	3.0E+04
USA 6	2.6E+10	2.0E+11	1.2E+10	1.2E+06	3.8E+11	4.0E+11	8.0E+09	5.0E+08

## Discussion

CT-PA bacteriophage cocktail displayed suitable anti-biofilm activity *in vitro*. It had a broad host range in the 45 isolates tested, with 89% of isolates susceptible. The use of a cocktail as opposed to individual phages increased the host range significantly, with only 53–73% of isolates being susceptible to each of the four phages individually. This is consistent with previous observations that the use of a cocktail of phages rather than individual phages improves activity by expanding the host range and by preventing the development of bacteriophage-insensitive mutant bacteria (Chan and Abedon, 2012; Hall et al., 2012; Drilling et al., 2014b).

The lytic effect of CT-PA on planktonic bacteria translated well to a reduction in biofilm, consistent with previous reports showing efficacy of bacteriophage to reduce biofilm *in vitro* and *in vivo* (Drilling et al., 2014a,b). Moreover, the increase in phage titres following treatment for almost all isolates implies successful phage infection, replication, and lysis of host bacteria, given the strictly lytic nature of all phages comprising the CT-PA phage cocktail.

Our results suggested slightly more biofilm removal by 10^7^ PFU/mL compared to 10^8^ PFU/mL CT-PA after 48 h treatment, despite similar anti-biofilm activity of the two treatment concentrations at 24 h. Studies of other *P. aeruginosa* phages have demonstrated that a higher concentration of phage treatment does not always result in greater magnitude of biofilm removal or bacterial killing (Knezevic et al., 2011; Worley-Morse et al., 2014). A non-linear relationship may be expected for some phage combinations over time due to the self-replicating nature of phage therapy (Abedon, 2011).

The isolates in our study sample consisted of 44 MLST sequence types from both the sinuses of non-CF CRS sufferers, and the upper and lower respiratory tracts of CF patients. BURST and phylogenetic analysis was consistent with the predominantly non-clonal population structure that has been described by *P. aeruginosa* strain typing studies (Kiewitz and Tümmler, 2000; Curran et al., 2004). Clonal groups (identical ST) and clonal complexes were uncommon within our collection of isolates. The clonal and BURST groups identified contain isolates from both CF and non-CF CRS patients, suggesting that *P. aeruginosa* sinus infections in both groups may initially be caused by similar strains. This is consistent with the observation made by Cramer et al that the dominant clones amongst CF patients are also common in the environment and other human disease habitats (Cramer et al., 2012).

However, frequent antibiotic resistance was displayed by CF isolates, with 52% of the CF strains tested meeting the criteria for multidrug-resistance. Rates of antibiotic resistance in non-CF CRS isolates were much lower, and comparable to those observed in similar studies of CRS patients and in data from national antimicrobial surveillance programs (Kingdom et al., 2004; Sasikala and Sundararaj, 2012; Sader et al., 2014, 2017; AURA, 2016). Higher rates of antibiotic resistance in *P. aeruginosa* isolates from CF patients compared to non-CF patients have been well-documented (Doring et al., 2000; Henwood et al., 2001). Although some of the isolates in our study were from the lower respiratory tract of CF patients, a high degree of similarity in the adaptive mutations and gene expression of CF sinus and lung *P. aeruginosa* isolates has been found (Ciofu et al., 2013). Furthermore, the paranasal sinuses have been found to be a reservoir of recurrent pulmonary infections in CF patients, with identical strains often identified in the upper and lower respiratory tracts of patients (Roby et al., 2008; Johansen et al., 2012; Aanaes, 2013; Aanaes et al., 2013). Thus, the selective pressures in the upper respiratory tract of CF patients, including antibiotics administered for management of bacterial sinus infections, have potential implications for the development of chronic lung infections (Hansen et al., 2012). The progression to chronic *P. aeruginosa* lung infection in CF has been shown to lead to deterioration in lung function and decreased life expectancy (Mastella et al., 1999; Li et al., 2005).

The proportion of clinical isolates that were resistant to lysis by the CT-PA phage cocktail was small (11%), and did not include any multidrug resistant isolates. Importantly, the anti-biofilm activity of CT-PA was not affected by multidrug resistance, or whether the isolate was from a CF patient. The CF isolates tested were largely comprised of isolates with a small colony or mucoid phenotype. These phenotypes are associated with chronic infection, and display increased tolerance to various antibiotics (Nichols et al., 1988; Govan and Deretic, 1996; Haussler et al., 1999; Guss et al., 2009; Hansen et al., 2012). These findings suggest that phage therapy may have potential in treating CF patients with chronic *P. aeruginosa* infection, for whom clinicians are currently faced with a dilemma of how to treat respiratory infections without creating further antibiotic resistance. We have previously demonstrated the ability of high-volume nasal irrigations to penetrate into the paranasal sinuses following functional endoscopic sinus surgery (FESS). (Grobler et al., 2008) The phage cocktail could be administered via nasal irrigations in order to treat recurrent sinus infections following FESS.

*In vivo* animal studies have demonstrated enhanced bacterial clearance and prolonged survival in phage-treated animals (in *Galleria mellonella*, canine otitis, murine lung, corneal and peritoneal *P. aeruginosa* infection models) (Heo et al., 2009; Debarbieux et al., 2010; Hawkins et al., 2010; Alemayehu et al., 2012; Fukuda et al., 2012; Beeton et al., 2015). A limited number of early-stage clinical trials of phage therapy for treatment of *P. aeruginosa* infections have been undertaken. A double-blind randomized controlled trial of a topically-applied phage cocktail for antibiotic-resistant *P. aeruginosa* chronic otitis showed statistically significant improvement from baseline in both symptom and clinical severity scores in the phage treatment group (Wright et al., 2009). No such improvement was observed in the placebo group. Conversely, two clinical trials of phage cocktails targeting multiple bacterial pathogens (*P. aeruginosa, S. aureus*, and *E. coli*) for treatment of burns and chronic leg ulcers, respectively showed no significant differences between phage and control therapies (Rhoads et al., 2009; Rose et al., 2014).

The aforementioned clinical trials all reported excellent safety profiles. A study of long-term safety of a *S. aureus* phage cocktail applied topically to sheep sinuses found no local or systemic safety concerns (Drilling et al., 2017). Although not widespread, clinical use of phage therapy has occurred through phage therapy and research institutes, such as the Eliava Institute in Tbilisi, Georgia, and the Institute of Immunology and Experimental Therapy in Wroclaw, Poland (Sulakvelidze et al., 2001; Kutateladze, 2015). An analysis of 153 patients treated for various infections with phage therapy at the latter institute revealed no major safety concerns (Międzybrodzki et al., 2012).

Whilst further evaluation and optimisation of phage cocktail treatment of *P. aeruginosa* infections in CRS and CF are required, the safety profile of phage therapy and its activity against antibiotic-resistant isolates make it an attractive potential therapeutic option. Further pre-clinical and clinical studies will be of great benefit in determining whether phage therapy can fulfill the potential demonstrated in this study.

## Author contributions

SF contributed to study design, execution of experiments, data analysis, and writing of manuscript. AD contributed to study design, execution of experiments, data analysis, and review of manuscript. SM and SV contributed to study design, and review of manuscript. MC assisted with collection of clinical samples, and data collection. BW and WF contributed to collection of clinical samples, and review of manuscript. AP and PW contributed to study design, collection of clinical samples, and review of manuscript.

### Conflict of interest statement

SM is an employee of AmpliPhi Australia, a biotechnology company focused on the development of bacteriophage products. The other authors declare that the research was conducted in the absence of any commercial or financial relationships that could be construed as a potential conflict of interest
