# Supplementary material for: Activity of Bacteriophages in Removing Biofilms of Pseudomonas aeruginosa Isolates from Chronic Rhinosinusitis Patients
Source: Front Cell Infect Microbiol. 2017 Sep 22;7:418. doi: 10.3389/fcimb.2017.00418 (PMC5615211; doi:10.3389/fcimb.2017.00418)
Supplement: Supplementary file 1 [file Table1.DOCX]

Supplementary Material

Activity of Bacteriophages in Removing Biofilms of *Pseudomonas aeruginosa* Isolates from Chronic Rhinosinusitis Patients

**Stephanie A. Fong, Amanda Drilling, Sandra Morales, Marjolein E. Cornet,**

**Bradford A. Woodworth, Wytske J. Fokkens, Alkis J. Psaltis, Sarah Vreugde and**

**Peter-John Wormald**

*** Correspondence:**

Peter-John Wormald, MD

Phone: +618 82227158

Email: peterj.wormald@adelaide.edu.au

# Supplementary Figures and Tables

## Supplementary Table 1: MLST profiles of *P. aeruginosa* strains used in this study

ST: MLST sequence type

a, b, c: Clonal groups (identical sequence type)

d: Clonal complex (6 alleles in common)

e, f, g: BURST group isolates (5 alleles in common)

| **Isolate** | **Country of origin** | **ST** | **acsA** | **aroE** | **guaA** | **mutL** | **nuoD** | **ppsA** | **trpE** |
| --- | --- | --- | --- | --- | --- | --- | --- | --- | --- |
| PA01 | Lab reference | 549 ^a f^ | 7 | 5 | 12 | 3 | 4 | 1 | 7 |
| P1 | Australia | 348 ^b^ | 22 | 20 | 11 | 3 | 3 | 3 | 7 |
| P2 | Australia | 446 | 18 | 4 | 5 | 13 | 1 | 17 | 13 |
| P3 | Australia | 910 | 34 | 5 | 20 | 31 | 1 | 15 | 10 |
| P4 | Australia | 155 | 28 | 5 | 36 | 3 | 3 | 13 | 7 |
| P5 | Australia | 242 | 28 | 5 | 5 | 11 | 3 | 15 | 44 |
| P6 | Australia | 1517 | 11 | 5 | 30 | 3 | 3 | 38 | 7 |
| P7 | Australia | 274 ^c^ | 23 | 5 | 11 | 7 | 1 | 12 | 7 |
| P8 | Australia | 17 ^g^ | 11 | 5 | 1 | 7 | 9 | 4 | 7 |
| P9 | Australia | 348 ^b^ | 22 | 20 | 11 | 3 | 3 | 3 | 7 |
| P10 | Australia | 395 | 6 | 5 | 1 | 1 | 1 | 12 | 1 |
| P11 | Australia | 643 ^f^ | 28 | 5 | 12 | 3 | 4 | 1 | 18 |
| P12 | Australia | 499 | 11 | 5 | 7 | 27 | 2 | 7 | 33 |
| P13 | Australia | 642 | 125 | 105 | 36 | 3 | 3 | 15 | 2 |
| P14 | Australia | 988 | 16 | 5 | 36 | 3 | 4 | 7 | 37 |
| P15 | Australia | 399 | 11 | 5 | 1 | 2 | 2 | 15 | 2 |
| P16 | Australia | 266 | 16 | 5 | 11 | 72 | 44 | 7 | 52 |
| P17 | Australia | 386 | 17 | 5 | 11 | 18 | 4 | 10 | 3 |
| P18 | Australia | 270 | 22 | 3 | 17 | 5 | 2 | 10 | 7 |
| P19 | Australia | 1637 | 11 | 5 | 3 | 3 | 8 | 1 | 9 |
| P20 | Australia | 1399 | 28 | 10 | 1 | 3 | 27 | 4 | 7 |
| P21 | Australia | 527 | 16 | 52 | 11 | 85 | 59 | 15 | 10 |
| A01 | Netherlands | 575 | 11 | 5 | 83 | 2 | 4 | 13 | 7 |
| A03 | Netherlands | 1221 | 89 | 30 | 64 | 90 | 48 | 24 | 32 |
| A10 | Netherlands | 274 ^c^ | 23 | 5 | 11 | 7 | 1 | 12 | 7 |
| A14 | Netherlands | 1684 ^e^ | 39 | 6 | 9 | 11 | 3 | 3 | 2 |
| A17 | Netherlands | 511 | 6 | 85 | 1 | 5 | 1 | 4 | 68 |
| A23 | Netherlands | 549 ^a f^ | 7 | 5 | 12 | 3 | 4 | 1 | 7 |
| B08 | Netherlands | 584 | 17 | 5 | 5 | 18 | 4 | 12 | 83 |
| B12 | Netherlands | 559 | 11 | 5 | 77 | 5 | 3 | 6 | 68 |
| B14 | Netherlands | 406 | 40 | 5 | 11 | 3 | 4 | 13 | 7 |
| B18 | Netherlands | 164 | 1 | 5 | 1 | 11 | 4 | 10 | 10 |
| B20 | Netherlands | 1225 | 118 | 106 | 85 | 92 | 60 | 54 | 72 |
| B22 | Netherlands | 245 ^e^ | 39 | 6 | 12 | 11 | 3 | 15 | 2 |
| B24 | Netherlands | 497 ^g^ | 11 | 5 | 1 | 7 | 3 | 56 | 7 |
| C2 | Netherlands | 581 | 39 | 5 | 20 | 5 | 1 | 67 | 31 |
| C4 | Netherlands | new | 17 | 1 | 11 | 11 | 4 | 4 | 7 |
| C5 | Netherlands | new | 124 | 5 | 6 | 3 | 4 | 4 | 189 |
| C7 | Netherlands | 558 | 40 | 22 | 1 | 3 | 2 | 6 | 7 |
| C10 | Netherlands | 485 | 11 | 76 | 5 | 3 | 61 | 14 | 3 |
| C12 | Netherlands | 492 | 40 | 5 | 30 | 61 | 1 | 7 | 14 |
| UAB 1-1 | USA | new ^d^ | 164 | 3 | 7 | new | 2 | 4 | 19 |
| UAB 1-2 | USA | new ^d^ | 164 | 3 | 7 | new | 2 | 12 | 19 |
| UAB 2 | USA | 274 ^c^ | 23 | 5 | 11 | 7 | 1 | 12 | 7 |
| UAB 3 | USA | 27 | 6 | 5 | 6 | 7 | 4 | 6 | 7 |
| UAB 4-2 | USA | new | 15 | 5 | 11 | 3 | 4 | 42 | 9 |
| UAB 5 | USA | 179 | 36 | 27 | 28 | 3 | 4 | 13 | 7 |
| UAB 6 | USA | new | 99 | 5 | 30 | 67 | 3 | 33 | 14 |
